# Decoding the immune landscape in Ewing sarcoma pathogenesis: The role of tumor infiltrating immune cells and immune milieu

**DOI:** 10.1016/j.jbo.2025.100678

**Published:** 2025-03-31

**Authors:** Rajiv Ranjan Kumar, Nikita Agarwal, Akshi Shree, Jaya Kanta Gorain, Ekta Rahul, Shuvadeep Ganguly, Sameer Bakhshi, Uttam Sharma

**Affiliations:** a Department of Medical Oncology, Dr. B.R.A. Institute Rotary Cancer Hospital, All India Institute of Medical Sciences, New Delhi, India; b Department of Biomedical Science, Shaheed Rajguru College of Applied Sciences for Women, University of Delhi, Delhi 110096, India; c Department of Pathology, Atal Bihari Vajpayee Institute of Medical Sciences and Dr. Ram Manohar Lohia Hospital, Delhi 110001, India; d Department of Medical Oncology, Jawaharlal Institute of Postgraduate Medical Education & Research, Puducherry, India

**Keywords:** Tumor microenvironment, Tumor immune microenvironment, Ewing sarcoma, Immunotherapy, Immune infiltration

## Abstract

Ewing sarcoma (EwS) is the second most prevalent pediatric bone malignancy, characterized by its aggressive behavior and unfavorable prognosis. The tumor microenvironment (TME) of EwS is shaped by immunosuppressive components, including myeloid-derived suppressor cells, tumor-associated macrophages, and immune checkpoint molecules such as PD-1/PD-L1 and HLA-G. These elements impair anti-tumor immune responses by modulating the function of tumor-infiltrating immune cells, such as regulatory T cells (Tregs), CD8^+^ T cells, and natural killer cells. Chemokines, including CXCL9 and CXCL12, and cytokines, such as transforming growth factor-beta and interleukin-10, further contribute to immune suppression and promote metastatic dissemination. Recent advances in immunotherapy have highlighted the therapeutic potential of modulating immune cells and signaling pathways to enhance anti-tumor immunity. This review provides a comprehensive analysis of the complex immune landscape within the EwS TME, focusing on the mechanistic roles of key immune components and their potential as therapeutic targets. Understanding these interactions could pave the way for innovative treatment strategies to improve clinical outcomes in patients with EwS.

## Highlights

• Immune cells, including T cells, NK cells, and TAMs, critically influence the tumor microenvironment in EwS.
• Regulatory T cells and M2-polarized TAMs promote immunosuppression, whereas NK cells provide potential for direct tumor cell elimination.
• Tumor-derived molecules such as PD-L1, PD-L2, and HLA-G suppress immune cell activity, facilitating immune evasion and tumor proliferation.
• Cytokines like TGF-β and IL-10, along with chemokines such as CXCL12, establish an immunosuppressive microenvironment that promotes metastasis and tumor progression.
• Emerging therapies, including tyrosine kinase inhibitors, PARP inhibitors, and cyclin-dependent kinase inhibitors, demonstrate significant promise in targeting EwS.
• Innovative immunotherapies, such as CAR T cell therapies targeting GD2 and EGFR, NK cell-based treatments, and TCR-transduced T cells, are under active investigation, representing cutting-edge approaches to enhance immune-mediated tumor control in EwS.

## 1 Introduction

Ewing sarcoma (EwS) is the second most prevalent pediatric bone malignancy, characterized by its aggressive behavior and unfavorable prognosis. Based on histology, EwS tumor cells clustered in sheets with finely dispersed chromatin and a high nucleus-to-cytoplasm ratio [1]. EwS is driven by the t(11;22)(q24;q12) translocation, fusing EWSR1 (chromosome 22) with FLI1 (chromosome 11) to produce the oncogenic EWS-FLI1 fusion protein [2].

Despite the availability of several multimodal treatments, such as systemic chemotherapy combined with local control achieved by radiation or surgery, the 5-year survival rate for metastatic EwS is still quite low (∼10–30 %). Intensive therapeutic strategies for EwS are now exploring targeted approaches, including histone deacetylase inhibitors, small molecule inhibitors like YK-4-279, and lysine-specific histone demethylase 1 (LSD1) inhibitors to improve patients’ outcomes [3], [4], [5], [6]. Due to the limited efficacy of traditional treatments, there is an increasing demand to assess new therapeutic targets or strategies to enhance EwS patients’ prognosis in clinical settings. Researchers are exploring the tumor immune microenvironment (TIME) to understand tumor evolution and identify novel treatment options for Ewing sarcoma (EwS). Therapeutic strategies under investigation include monoclonal antibodies, chimeric antigen receptor (CAR) T-cell therapies, cancer vaccines, oncolytic viruses, immune checkpoint inhibitors, and cytokine-based therapies are being explored, all designed to enhance the body's anti-tumor immune response [4], [7], [8], [9], [10]. In addition, varying numbers of tumor-infiltrating lymphocytes (TILs) play a role in maintaining the TME in Ewing's sarcoma family of tumors (ESFT) [2]. In a study by Berghuis *et al.* (2011), a follow-up over 100 months demonstrated that patients with high CD8+tumor-infiltrating lymphocyte (TIL) expression had an overall survival rate of 90 %, compared to those with low CD8+TIL expression. This finding underscores the potential of targeting TILs and modulating the TME to enhance long-term survival in EwS patients [11].

Furthermore, expression profiling of inflammatory chemokines and their receptors in EwS, including C-X-C motif chemokine receptor 1 (CXCR1), C-X-C motif chemokine receptor 3 (CXCR3), and C-C motif chemokine receptor 5 (CCR5), as well as Interferon Gamma (IFNγ) and C-X-C motif chemokine ligand 9/10 (CXCL9/10), etc has demonstrated a strong association with the TME. These factors play an important role in recruiting CD8+T-cells for immune infiltration to EwS TME, contributing to improved overall survival of EwS patients and enhanced response to immunotherapy **(**Table 1**)** [1], [3], [12], [13], [14]. Notably, activation of CCL21, the ligand for chemokine receptor CCR7, is associated with the CD4+/CD8+ratio of infiltrating T-cells and clinical outcomes, including improved chemotherapeutic response and prognosis, suggesting its potential as a prognostic marker [14], [15], [16]**.** The tumor-infiltrating T-cells, as well as CAR gene-modified effectors, also play an important role in providing response to EwS cells, which upregulates HLA-G expression, suggesting the higher efficacy of cellular immunotherapies in EwS **(**Table 1**)** [17], [18]**.** There have been several reports wherein the presence of a high white blood cell count has been observed to be a poor prognostic marker in this disease, and this signifies the role of inflammation in this cancer [19], [20], [21]. Besides these, different immune cells, including Natural Killer (NK), mast, B, and Th2 cells, were also reported in EwS, suggesting a role in pathogenesis that may help develop prognostic biomarkers **(**Table 1**)** [19], [22].

**Table 1:** Affected immune cells and molecules and their biological function in Ewing Sarcoma.

S.No.	**Sample types (peripheral blood, tissues, cell lines, animal model)**	**Clinical samples**		**Cell lines name**	**Tumor infiltrating cells and molecules**	**Molecular targets of signaling pathway**	**Affected Signaling pathways**	**Biological function**	**Techniques involved**	**References**
		**Patients (n)**	**Control (n)**							
1	Peripheral blood, tumor tissue, cell lines, animal model	Peripheral blood (n = 19); Tumor tissue (n = 47)	Peripheral blood (n = 15); Tumor tissue (n = 12)	VH-64, TC-32, A4573, 5838, TTC-466, RD-ES, TC-71, SK-ES1, CADO-ES-1, MS-PES-1, MS-PES-4, MS-PES-6, DC-ES-6, K562 and JEG-3	HLA-G, T-cells and NK cells, KIR2DL4, ILT2, ILT4, INF-ƴ	HLA-G, INF-ƴ	INF-ƴ stimulating pathway	Immune suppression and escape	ELISA, Western blot, immunohistochemistry (IHC), Flow cytometry	[18]
2	Tumor tissue, cell lines	n = 18	n = 16	L-1062, L-872, CHP100, RM-82, IARC-EW7, TC32, 6647, CHP100, RM-82, IARC-EW-7, WE-68, IARC-EW-3, STA-ET-2.1, TTC-466, STA-ET-10, CADO-ES1, TC-71, VH-64, COH, STA-ET-1, SK-ES-1, SK-NM-C, A-673 and R-D-ES	CCL21, CD4+T-Cells, CD8+T-Cells, NK cells, dendritic cells, CXCL9, CXCL10, IFN-ƴ	CCL21, CXCL9, CXCL10, IFN-ƴ	CCL21-CCR7 expression	Pro-inflammatory chemokines expression	RT-qPCR, IHC	[14]
3	Database Analysis	Training set (n = 88)	Validation set (n = 57)	NA	CRLF3, ECD, FABP4, FGF6, GNRH2, NDRG1, PAK2, PLTP, PTGDS, RBP1, ZC3HAV1, B-cells, CD8+T, NK cells, Th2, cytotoxic T-cells, macrophages, mast cells, VEGFA, MMP9, CXCL8, EGF, IGF1, CXCR4, TGFB1, EGFR, SPP1, and ICAM1	SOX2, STAG2, TP53, CXCR4	NA	Tumor cell proliferation and angiogenesis	AI	[22]
4	Peripheral blood, cell lines, animal model	NA	NA	RD-ES, A673	NK cells, USP6, integrin LFA-1, MIC-A/B, CD112, CD155, JAK1, IFN receptors	USP6, IFN-γ, CXCL9, CXCL10, JAK1	Jak1/STAT1 Pathway, Type I and Type II Interferon Receptors	Induction of IFN response genes, including CXCL12; NK cell recruitment, maturation and activation	NK Cytotoxicity Assays, Flow cytometry, RT-qPCR	[39]
5	Peripheral blood	n = 17	n = 8	NA	TIM 3, Galectin 9, PD-1, LAG-3, T-cell	TIM-3, Galectin-9, PD-1, LAG-3	PD-1 pathway, TIM-3/Galectin-9 axis	T-cell inhibition	Flow cytometry, IHC, Cell culture	[51]
6	Database Analysis	Training set (n = 179)	Validation set (n = 57)	NA	T-cell, FMO2, GLCE, GPR64, IGFBP4, LOXHD1, SNAI2, SPP1, TAPT1-AS1, ZIC2	miR205-5p, PGF, MMP1, SNAI2, c-MET, PD-L1, STAT3	STAT pathway, PD-L1 Pathway	Tumor angiogenesis, invasion and metastasis	Survival analysis	[58]
7	Peripheral blood, cell lines	Cell lines (n = 5)	Healthy donors numbers not specified	A4573, A673, TC32, TC71, MRC5	TNF, IL-1α, IL-1β, IL-6, IL-8, CCL2/MCP1, CCL3/MIP −1α, CCL4/MIP-1β	TLR2-MyD88, IL-6, IL-8, CD47, PD-L1	TLR signaling pathways, type I/III interferon responses, cytokine release pathways, and immune checkpoint pathways	Pro-inflammatory function	Cytokine Single- and Multiplex Assay, Flow Cytometry, Whole Transcriptome Sequencing, Bioinformatics	[35]
8	Tumor tissue	n = 11	NA	NA	pDC, cDC1 and cDC2	NRP1/2-VEGFA, SPP1-CD44, SEMA4D-PLXNB2, NRP1-SEMA3A, LRP1-MDK, IGF1-IGF1R, FPR1/2/3-ANXA1, TNFSF10-TNFRSF10B (TRIAL-TRIAL-R), SIRPA-CD47, C5AR1-RPS19, and GAS6-AXL	NFκB and STAT3 signaling pathway	Immune evasion, immunosuppressive tumor microenvironment	scRNA-seq, Flow cytometry	[31]
9	Tumor tissue, cell lines	NA	NA	A673, TC-71, VH-64, A4573, TC-32, K562, THP-1	HLA-G, HLA-E	IFN-γ stimulation	INF-ƴ stimulating pathway	Inhibit the antigen-specific effector functions of CART, IFN-γ cytokine stimulation	Western Blot, ELISA, Flow cytometry, CRISPR/Cas9, IHC	[17]
10	Database Analysis, cell lines	n = 173	NA	RD-ES, A673	ANXA1, COL1A2, MMP9, VIM, S100A11, S100A4, HAVCR2, CSF1R, IL-10, LGALS9, IL10RB, PDCD1LG2, TGFB1, TGFBR1, TIGIT and CD96	ANXA1	Immune related pathway	Mediating inflammatory responses, proliferation, metastasis and drug resistance	RT-qPCR, CCK-8, EdU assay	[69]
11	Database Analysis, cell lines	n = 106	NA	RD-ES, A673	CTSD, SIRPA, FN1, MDSCs, NK cell	CTSD- C5a/C5aR1, SIRPA- CD47 ligand; FN1- M2 macrophage polarization	Immune-related pathways, Akt and MAPK signalling pathway	Inhibit the recruitment of M2 macrophages, increase migration and phagocytosis of macrophages to exert antitumor effects	Western blot, RT-qPCR, IHC	[48]
12	Tumor tissue, cell lines, animal model	NA	NA	RD-ES, A673, SK-N-MC, TC-71	USP6, CXCL9, CXCL10, CCL5	USP6, IFN-γ, CXCL9, CXCL10	Chemotaxis and chemokine signaling, JAK-STAT pathway	Induction of ligands for CXCR3 and CCR5, increased cell migration	Dual Luciferase Assay, ELISA, Flow cytometry Chemokine antibody array, Migration assays	[3]
13	Tumor tissue, peripheral blood	n = 46	n = 10	NA	TIN, NET, CXCL1, IL-7, IL-8, MIP-1α, soluble CD40 ligand, CXCL5, TPO	G-SCF, IL-8	Immune-related pathways	NETs shield tumor cells from cytotoxic immune cells, specifically CD8+T-cells and NK cells, resulting in impaired tumor clearance	Immunofluorescent staining, NE-DNA complex ELISA	[1]
14	Database Analysis, cell lines	n = 88	n = 18	A673, RD-ES	PUS1, COX6A2, NDUFB9, NDUFB10, SLC25A12, PPM1B, RYR1	NDUFB10- SRC kinase, SLC25A12, COX6A2-COX subunit, SLC25A12	Oxidative phosphorylation, aerobic glycolysis, immune regulation pathways, IL6/JAK/STAT3 signaling, and specific developmental signaling pathways like hedgehog and notch signaling, PPM1B- p38-RB1-E2F1 pathway	Mitochondrial dysfunction and inhibit mitochondrial respiration in tumor cells, which would in turn facilitate the shift from oxidative phosphorylation to aerobic glycolysis resulting in increased lactate secretion.	RT-qPCR	[46]
15	Database Analysis	Immune cell types using 197 microarray gene expression (n = 22)	NA	NA	M2 macrophages, T-cells, activated NK cells, HIF-1α, EZH2, PD-L1, IL-10, TGF-β, ROS, IGFR1, OX40	HIF-1α	HIF-1α signaling pathway, Immune-related pathways, oxidative phosphorylation pathway	HIF1α is responsible for regeneration and tissue repair, increased myeloid-derived suppressor cells and polarization toward M2 macrophages.	Survival Analysis	[2]
16	Database Analysis, cell lines	n = 33	Not given	A673, SK-N-MC	FOXP4-AS1	miRNAs, mRNAs, TMPO, FOXP4-AS1	E2F targets, G2M checkpoints, MYC targets V1, epithelial-mesenchymal transition, p53 cascade, glycolysis, mTORC1 signaling, DNA repair, and protein secretion	Cell proliferation, migration and invasion	RT-qPCR, cell proliferation migration, invasion assay, western blot TEM	[64]
17	Database Analysis, cell lines	n = 85	n = 57	RD-ES, SK-ES-1, A673, SK-N-MC, hBMSCs	IL1B, IL11, ARAP3, PODXL, MDSCs, macrophages, Treg, IL6, CCL5, CXCL12, TGFB3, CSF1, RANKL (TNFSF11)	ARAP3, MDM2, p21, Bax, p53	p53 pathway, PI3K-Akt pathway	Tumor cell proliferation, survival, metastatic processes and angiogenesis	RT-qPCR, Western Blot, IHC, Cell proliferation assay, Flow cytometry, migration assay	[63]
18	Peripheral blood, tumor tissue	n = 200	n = 600	NA	CD68+, CD8+T-cells, macrophages, CD14 + CD16- and CD14 + CD16 + monocyte, SELL, CCR7, GZMB, KLRG1, KLFB1, PRF1, LAG3, PDCD1, IFNG, IFITM1, IFITM2, IFITM3, MKI67, ICOS, CTLA4, TIGIT, HAVCR2, GZMA, PRDM1, ITM2C, ITGA1, CXCR3, LGALS3, SOCS3, C1QA, C1QB, C1QC, RNASE1, FOLR2, MT1G, MT1X, HLA	CCL8, CCL3, CCL2, CXCL5, CXCL3, CXCL1, CCL7, CCL2, CXCL10, CXCL12 ligand	Immune-related pathways	Impair IFN- γ secretion from CD8+T cells, thus limiting antitumor immunity	Immunofluorescence staining, scRNAseq transcriptomics	[62]
19	Cell lines (A673, TC71) animal model	NA	NA	A673, TC71	M1(CD11c+/ CD206-) and M2 (CD11c+/CD206 + ) like macrophages, NK cells, MDSCs	IFN-γ, LPS, IFN + LPS, IL-10, IL-4, TGF-β	TGF- β signalling, immune checkpoint inhibition signalling	Inhibition of T-cell activation	Flow cytometry, PCR	[45]
20	Cell lines, animal model	NA	NA	ES1, ES2, ES4, ES6, ES7, ES8, SKNEP1, TC71, CHLA258, EW8	HGF, IFN-γ, IL-2, TNF-α, GD2	c-MET, Δ133p53 (p53 isoform)	HGF/c-MET pathway	Angiogenesis, cellular invasion, tumor growth and metastasis	RT-qPCR, Western blot, IHC, Quantitative ELISA, shRNA generation, Proliferation assay, Flow cytometry	[74]

**Abbreviations: KIR2DL4** − killer cell immunoglobulin-like receptor 2DL4; **ILT2**- immunoglobulin-like transcript 2; **ILT4**- immunoglobulin-like transcript 4, **INF-γ**- Interferon gamma; **HLA-G**- Human Leucocyte antigen G; **CCL21**- C–C Motif Chemokine Ligand 21; **CD**- Cluster of Differentiation; **CXCR**- C-X-C motif chemokine receptor; **CXCL**- C-X-C motif chemokine ligand; **CRLF3**- Cytokine Receptor Like Factor 3; **ECD**- Ecdysoneless Cell Cycle Regulator, **FABP4**- Fatty Acid Binding Protein 4, **FGF6**- Fibroblast Growth Factor 6; **GNRH2**- Gonadotropin Releasing Hormone 2; **NDRG1**- N-Myc Downstream Regulated 1; **PAK2**- P21 (RAC1) Activated Kinase 2; **PLTP**- Phospholipid Transfer Protein; **PTGDS-** Prostaglandin D2 Synthase; **RBP1**- Retinol Binding Protein 1; **ZC3HAV1**- Zinc Finger CCCH-Type Containing, Antiviral 1; **Th2**- T-helper cell; **VEGFA**- Vascular Endothelial Growth Factor A; **MMP9**- Matrix Metallopeptidase 9; **EGF**- Epidermal Growth Factor; **IGF1**- Insulin Like Growth Factor 1; **TGFB1**- Transforming Growth Factor Beta 1; **EGFR**- Epidermal Growth Factor Receptor; **SPP1**- Secreted Phosphoprotein 1; **ICAM1**- Intercellular Adhesion Molecule 1; **SOX2**- SRY-Box Transcription Factor 2; **STAG2**- Stromal antigen; **TP53**- Tumor Protein P53; **USP6**- Ubiquitin Specific Peptidase 6; **LFA-1**- Lymphocyte function-associated antigen 1; **MIC-A/B**- MHC Class I Polypeptide-Related Sequence A/B; **JAK1**- Janus Kinase 1; **TIM 3**- T-cell immunoglobulin and mucin domain 3; **PD-1**- Programmed death-1; **LAG-3**- Lymphocyte Activating 3; **FMO2**- Flavin Containing Dimethylaniline Monoxygenase 2; **GLCE**- Glucuronic Acid Epimerase; **GPR64**- G coupled receptor 64; **IGFBP4**- Insulin Like Growth Factor Binding Protein 4; **LOXHD1**- Lipoxygenase Homology PLAT Domains 1; **SNAI2-** Snail Family Transcriptional Repressor 2; **SPP1**- Secreted Phosphoprotein 1; **TAPT1-AS1**- TAPT1 Antisense RNA 1; **ZIC2**- Zic Family Member 2; **PGF**- Placental Growth Factor; **PD-L1**- Programmed Death Ligand-1; **STAT3**- Signal Transducer And Activator Of Transcription 3; **TNF**- Tumor Necrosis Factor; **IL**- Interleukin; **MIP**- Major Intrinsic Protein Of Lens Fiber; **NRP**- Neuropilin 1; **SEMA4D-PLXNB2**- Semaphorin 4D- Plexin B1; **LRP1-MDK**- LDL Receptor Related Protein 1-Midkine; **FPR1/2/3-ANXA1**- Formyl Peptide Receptor 1/2/3-Annexin A1; **SIRPA**- Signal Regulatory Protein Alpha; **C5AR1-RPS19**- Complement C5a Receptor 1- Ribosomal Protein S19; **GAS6-AXL**- Growth Arrest Specific 6- AXL Receptor Tyrosine Kinase; **COL1A2**- Collagen Type I Alpha 2 Chain; **VIM**- Vimentin; **S100A11**- S100 Calcium Binding Protein A11; **S100A4**- S100 Calcium Binding Protein A4; **HAVCR2**- Hepatitis A Virus Cellular Receptor 2; **CSF1R**- Colony Stimulating Factor 1 Receptor; **LGALS9**- Galectin 9; **IL10RB**- Interleukin 10 Receptor Subunit Beta; **PDCD1LG2**- Programmed Cell Death 1 Ligand 2; **TIGIT**- T Cell Immunoreceptor With Ig And ITIM Domains; **CTSD**- Cathepsin D; **SIRPA**- Signal Regulatory Protein Alpha; **FN1**- Fibronectin 1; TIN- Tubulointerstitial Nephritis; **NET**- Neutrophil extracellular trap; **TPO**- Thyroid Peroxidase; **PUS1**- Pseudouridine Synthase 1; **COX6A2**- Cytochrome *C* Oxidase Subunit 6A2; **NDUFB9**- NADH:Ubiquinone Oxidoreductase Subunit B9/ B10; **SLC25A12**- Solute Carrier Family 25 Member 12; **PPM1B**- Protein Phosphatase, Mg2+/Mn2 + Dependent 1B; **RYR1**- Ryanodine Receptor 1; **HIF-1α**- Hypoxia Inducible Factor 1 Subunit Alpha; EZH2- Enhancer Of Zeste 2 Polycomb Repressive Complex 2 Subunit; **ROS**- Reactive Oxygen Species; **IGFR1**- Insulin Like Growth Factor 1 Receptor; **OX40**- Type 1 transmembrane glycoprotein; **Akt**- AKT Serine/Threonine Kinase; **MAPK**- Mitogen-Activated Protein Kinase; **NDUFB10**- NADH:Ubiquinone Oxidoreductase Subunit B10; **SRC**- SRC Proto-Oncogene, Non-Receptor Tyrosine Kinase; **SLC25A12**- Solute Carrier Family 25 Member 12; **COX6A2-COX** subunit- Cytochrome *C* Oxidase Subunit 6A2; **ARAP3**- ArfGAP With RhoGAP Domain, Ankyrin Repeat And PH Domain 3; PODXL- Podocalyxin Like; **CSF1**- Colony Stimulating Factor 1; **RANKL (TNFSF11)**- TNF Superfamily Member 11; **TMPO**- Thymopoietin; **FOXP4-AS1**- FOXP4 Antisense RNA 1; SELL- Selectin L; **GZMB**- Granzyme B, **KLRG1**- Killer Cell Lectin Like Receptor G1; **KLFB1**- Killer Cell Lectin Like Receptor B1, **PRF1**- Perforin 1; **LAG3**- Lymphocyte Activating 3; **PDCD1**- Programmed Cell Death 1; **IFNG**- Interferon Gamma; **IFITM**- Interferon Induced Transmembrane Protein 1; **MKI67**- Marker Of Proliferation Ki-67; **ICOS**- Inducible T Cell Costimulator; **CTLA4**- Cytotoxic T-Lymphocyte Associated Protein 4; **HAVCR2**- Hepatitis A Virus Cellular Receptor 2; **GZMA**- Granzyme A; **PRDM1**- PR/SET Domain 1; **ITM2C**- Integral Membrane Protein 2C; **ITGA1**- Integrin Subunit Alpha 1; **LGALS3**- Galectin 3; **SOCS3**- Suppressor Of Cytokine Signaling 3**;** C1QA- Complement C1q A/B/C Chain; **FOLR2**- Folate Receptor Beta; **MT1G**- Metallothionein 1G; **MT1X**- Metallothionein 1X; **HGF**- Hepatocyte Growth Factor; **GD2**- ganglioside, **LPS**- Lipopolysacchride.

Here, we aim to collate the intricate immune and molecular landscapes in EwS TME, focusing on tumor-infiltrating molecules, immune features, and immune cells. This review will strengthen our understanding of the co-evolution of tumor and immune systems in EwS patients.

## 2 Methodology

### 2.1 Search strategy

**Fig. 1:**
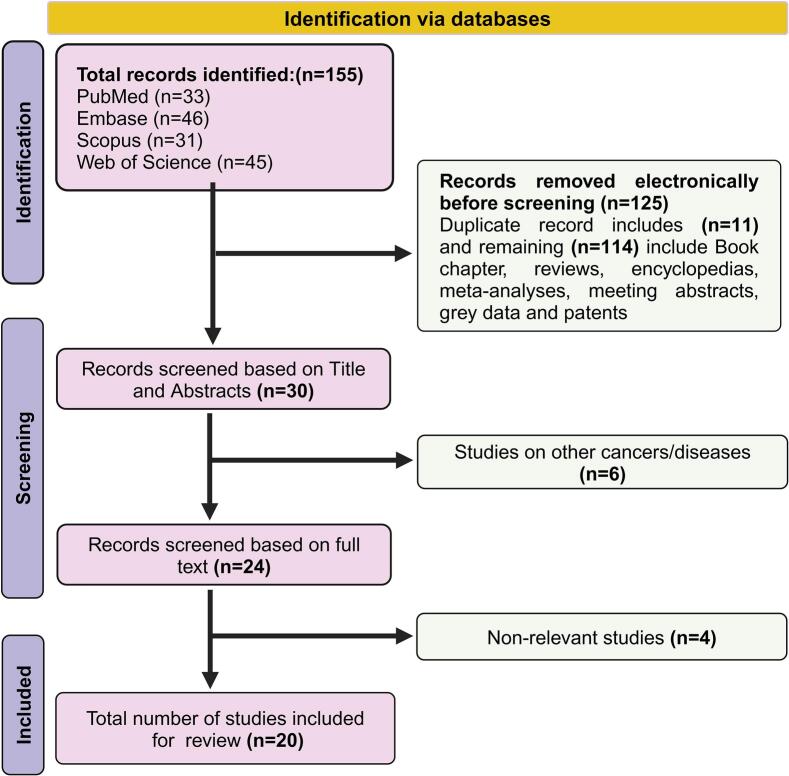
PRISMA flow chart outlining the literature search and study selection process.

We employed the preferred reporting items for systematic reviews and *meta*-analyses (PRISMA) guidelines for this review to retrieve and analyze relevant literature. The primary author (R.R.K) conducted the literature search with frequent consultations with a senior researcher (U.S). A comprehensive search was performed by using the keywords ((“Immune Infiltrate” AND (“Ewing Sarcomas”)); OR (“ES”)); OR (“PNET”)) OR (“EwS”)); AND (“Tumor microenvironment”)); OR (“TME”) AND (“Tumor immune microenvironment”) OR (“TIME”)). The systematic search was confined to publications from June 2014 to June 2024. Following the primary literature search, detailed information was sourced from several international online databases, including PubMed, Scopus, Embase, and Web of Science, as of 3rd December 2024. Studies were selected based on their title and abstracts. After a restrictive screening process, 155 articles were identified, as illustrated in Fig. 1.

### 2.2 Inclusion and exclusion criteria

This review exclusively focused on studies investigating the relationship between immune infiltration on TME, the tumor extracellular matrix (ECM), and the tumor immune microenvironment (TIME) in Ewing sarcoma. To maintain methodological integrity, we applied stringent inclusion criteria, considering only original research articles that specifically addressing immune infiltration in Ewing sarcoma. This approach ensures a focused and accurate analysis of the tumor immune microenvironment in EwS. An initial comprehensive web search identified several studies, among which duplicate records were excluded (n = 11) to eliminate redundancy. Furthermore, non-primary research materials were deemed ineligible and excluded, including reviews, book chapters, encyclopedias, meeting abstracts, meta-analyses, grey literature (e.g., user-generated content), and patents (n = 114). Articles that did not directly focus on Ewing sarcoma or were only indirectly related to the topic, such as those discussing other sarcoma subtypes or unrelated aspects of immune infiltration, were also excluded from this review (n = 10). Finally, 20 peer-reviewed original research articles met the inclusion criteria and were incorporated into the review. These selected studies provide critical insights into the intricate interactions between immune infiltration, ECM, and TIME in Ewing sarcoma, contributing to a deeper understanding of the tumor's immunobiology**.**

## 3 The implication of tumor-infiltrating cells and molecules in EwS

**Fig. 2:**
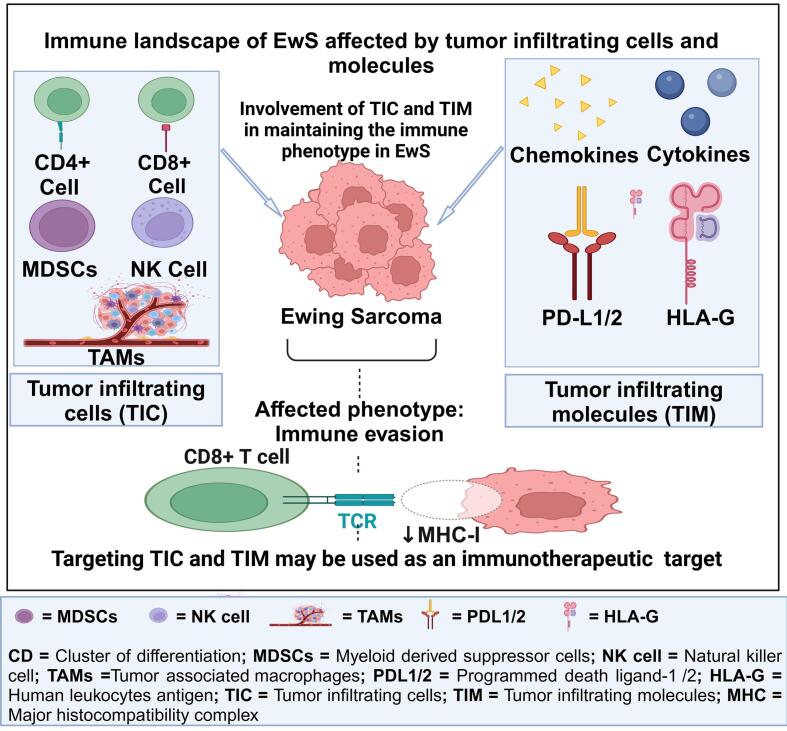
Immune landscape of EwS affected by tumor infiltrating cells and molecules.

The role of immune cells and molecules in immunotherapy and pathogenesis of EwS remains largely unknown. Myeloid-derived suppressor cells (MDSCs) and tumor-associated macrophages (TAMs) are immature monocytic subsets that influence the TME in EwS [23]. Their interactions with different immune molecules, including PD-L1, PD-L2, HLA-G, and tumor-infiltrating cells, such as T-cells, facilitate immunological escape in EwS [2], [24], [25]. Furthermore, other tumor-immune infiltrating molecules, such as chemokines, may contribute to developing therapeutic targets as they maintain local tumor dynamics and metastasis [4]. Still, their mechanism of action is not clear yet. Furthermore, the role of immunosuppressive cytokines in EwS is unresolved; however, immunosuppressive cytokines promote cell motility and invasiveness, which aids in the formation of tumors [26]. In further sections of this review, we described their mechanistic role in how cancer phenotypes are affected by immune infiltrating cells and molecules in EwS that may be used as therapeutic targets in EwS, as illustrated in Fig. 2**.**

### 3.1 Tumor infiltrating cells

Immune cells within the EwS TME adopt distinct roles, often shifting from their typical anti-tumor functions toward immune suppression and tumor support. Major immune cell types found in the TME include T-cells, NK cells, macrophages, and MDSCs, each contributing differently to the immune landscape. Tumor-infiltrating immune cells can be used as biomarkers and possible novel immunotherapeutic targets **(**Table 1**)** [2].

#### 3.1.1 T-Cells (CD4+ and CD8+)

**Fig. 3:**
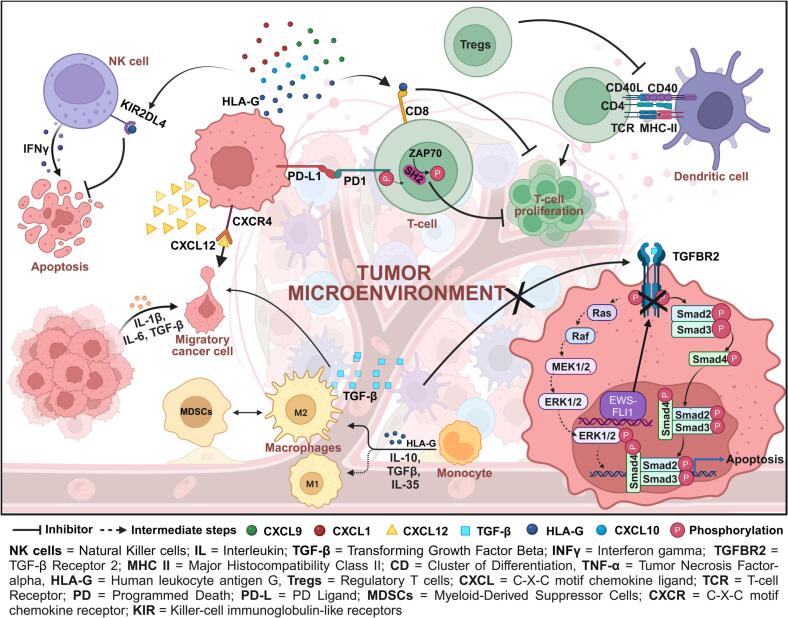
**Tumor infiltrating cells and molecules**: EwS creates an immune-suppressive microenvironment. TGF-β binds to the TGFBR2 receptor on tumor cells to induce apoptosis. However, the t(11;22) (q24;q12) chromosomal translocation develops the EWS-FLI1 fusion gene, which inhibits the interaction of TGF-β with TGFBR2 and prevents apoptosis. Additionally, HLA-G from tumor cells binds to KIR2DL4 (on NK cells) and CD8 (on T-cells), inhibiting apoptosis and T-cell proliferation. Regulatory T-cells (Tregs) are more active and hinder dendritic cell-mediated T-cell activation. M2 macrophages predominate, releasing TGF-β that promotes epithelial-to-mesenchymal transition (EMT), increasing cell motility. Tumor cells also release multiple chemokines (CXCL1, CXCL2, CXCL9, CXCL10, CXCL12), with CXCL12 interacting with its receptor CXCR4 on the tumor cells themselves, further aiding in cancer spread.

T-cells are essential for immune functions, maintaining health, and preventing disease. T-cell development occurs progressively in the thymus, leading to the formation of CD4+ and CD8+T-cell subsets. These subsets rapidly acquire effector functions, allowing them to eliminate infected cells and produce inflammatory cytokines necessary for immune responses [27], [28]. CD4+T-cells, also known as helper T-cells, are crucial for coordinating immune responses. They perform diverse roles, such as stimulating B lymphocytes, cytotoxic T-cells, non-immune cells, and elements of the innate immune system [29], as illustrated in Fig. 3. Furthermore, CD4+T-cells are essential for generating CD8+cytotoxic T lymphocyte (CTL) memory, emphasizing their role in adaptive immunity [30]. In EwS, the TME is defined by the presence of immunosuppressive cytokines, including transforming growth factor beta (TGF-β) and IL-10. These cytokines modulate CD4+T-cells, encouraging their differentiation into regulatory T-cells (Tregs), thereby contributing to an immunosuppressive milieu **(**Table 1**)** [29].

Tregs are a distinct subset of T cells for maintaining immune homeostasis and tolerance. They are key targets in tumor immunity research due to their multifaceted roles in modulating immune responses [4], [31], [32], [33]. In the EwS microenvironment, Tregs, along with other immunosuppressive cells like myeloid-derived suppressor cells (MDSCs) and F2 fibrocytes, suppress local antitumor immune responses **(**Table 1**)** [4]. Like other tumors, increased infiltration of Tregs is observed in EwS [34]. Tregs can suppress the activation and maturation of CD4+helper T-cells and CD8+cytotoxic T-cells, thereby limiting the immune response to tumor-expressed antigens. In EwS, the CD8+T and Treg populations exhibit tumor-associated T-cell signatures, while CD4+T-cells resemble normal Th17 and naïve T-cell profiles, reflecting a complex immune landscape [31], [33]. Focusing on the PD-L1/PD-1 axis has shown potential in reducing Treg expansion and improving T-cell functionality **(**Table 1**)** [33], [35]. PD-L1, a transmembrane protein and co-inhibitory factor, interacts with PD-1 to suppress the proliferation of PD-1-positive cells, block cytokine secretion, and trigger apoptosis [36]. However, in ESFTs, PD-L1 gene expression and several other immune-related molecules are often low or undetectable, presenting challenges for immunotherapeutic interventions [2].

#### 3.1.2 Natural killer (NK) cells

NK cells are cytotoxic innate lymphocytes critical for antitumor immunity. They target and destroy tumor cells by releasing cytotoxic granules that trigger apoptosis, playing a crucial role in the innate immune response [37]. In contrast to T-cells, NK cells engage with tumor cells without requiring antigen presentation, allowing them to circumvent major immune escape mechanisms often utilized by tumors [37]. NK cell responses are regulated by a balance between inhibitory and activating cell surface receptors, including NKG2D, natural cytotoxicity receptors, Ly49 or KIR, CD94–NKG2 heterodimers, and co-stimulatory receptors [38]. Mechanistically, low expression of MHC class I molecules and high expression of NK cell receptor activators in tumors suggest the potential utility of NK cells in immunotherapy, as depicted in Fig. 3.

In EwS, NK cells have a crucial role in suppressing tumor growth. The removal of NK cells negates the tumor-suppressive effects of ubiquitin-specific protease 6 (USP6) in EwS xenograft models, highlighting their significance in tumor immunity **(**Table 1**)** [39]. USP6 acts as a tumor suppressor in EwS and is the first recognized cell-intrinsic factor shown to influence the immune TME in this disease [39]. This interaction between USP6 and NK cells underscores potential opportunities for targeted therapeutic strategies [3]. Activated NK cells are linked to improved OS in cancer patients, further emphasizing their therapeutic potential [2], [40]. These findings suggest that leveraging NK cells, either directly through activation or indirectly by targeting modulators like USP6, could potentially enhance antitumor responses in EwS and other malignancies [39].

#### 3.1.3 Tumor-Associated Macrophages (TAMs)

Macrophages are highly adaptable immune cells pivotal in inflammation and regulation [41], [42]. Macrophages within the tumor microenvironment can adopt distinct functional phenotypes broadly classified as M1 (pro-inflammatory, anti-tumor) and M2 (anti-inflammatory, pro-tumor) phenotypes. M1 macrophages are activated by inflammatory stimuli, producing cytokines such as TNF-α and IL-12, which enhance immune responses and inhibit tumor growth. In contrast, M2 macrophages are associated with immune suppression, tissue remodeling, and tumor progression, secreting factors like IL-10, TGF-β, and VEGF, which promote angiogenesis and evade anti-tumor immunity [43], [44]. In EwS, the polarization of macrophages toward the M2 phenotype is influenced by interactions with various immune cells and signaling pathways, highlighting their critical role in shaping the tumor microenvironment and driving disease progression [44]. Macrophages within the tumor microenvironment that contribute to immunosuppression and promote tumor progression are known as tumor-associated macrophages (TAMs) [44]. TAMs predominantly adopt an M2 phenotype characterized by immunosuppressive and pro-tumor activities [42]. For example, Tregs heavily influence macrophage development by reducing IFN-γ production from CD8+T-cells, promoting an M2-like state **(**Table 1**)** [41]. Notably, M2-polarized TAMs are approximately 10 times more abundant than their M1 counterparts, emphasizing their significant role in tumor progression [45]. Zhang et al., suggested that M2 polarization is also facilitated by lactate through odor receptors and promotes tumor progression and immune escape **(**Table 1**)** [46]. Moreover, M2-polarized TAMs secrete various cytokines and growth factors that promote tumor growth, immune evasion, and suppression of cytotoxic immune cells [47], [48]. In EwS, the accumulation of M2-polarized TAMs within the TME contributes substantially to immunosuppression. These cells inhibit the activity of cytotoxic T-cells and impair the function of NK cells, reducing overall antitumor immunity [31], [49], [50], as illustrated in Fig. 3. Since the majority of TAMs in EwS likely adopt an M2 phenotype, supporting tumor progression. Therefore, we speculate that reprogramming M2-TAMs to M1-like states using CSF-1R inhibitors, TLR agonists, or IFN-γ therapy could enhance anti-tumor immune activity.

#### 3.1.4 Myeloid-derived suppressor cells (MDSCs)

Myeloid-derived suppressor cells (MDSCs) are a subset of immature monocytic and granulocytic cells [4] that originate from myeloid tissue and are comprised of myeloid cell progenitors, precursors of DCs, monocytes, macrophages, and granulocytes [23]. MDSCs can transform into TAMs and influence macrophage behavior within the TME. Like other cancers, TAMs are crucial for the development and progression of EwS. MDSCs promote macrophage polarization toward an anti-inflammatory (M2-like) phenotype in EwS by dysregulating STAT3 pathway [45]. These macrophages are typically recruited to the EwS TME, where they produce IL-10, suppressing macrophages' ability to produce IL-12, further reinforcing the M2 phenotype. Additionally, MDSCs express high levels of arginase-1, further driving M2 polarization and contributing to immune suppression [41], as shown in **[**Fig. 3**]**. MDSCs also release cytokines that inhibit the activity of cytotoxic T cells, similar to Tregs. Tregs, in turn, enhance the growth and suppressive functions of MDSCs through a TGF-β dependent mechanism [33]. Targeting MDSCs via STAT3 inhibitor or arginase-1 and CXCR4 or TGF-β antagonists could improve immune responses in EwS.

### 3.2 Immune molecules in tumor microenvironment

The interplay of the tumor-derived molecules like immune checkpoint ligands (PD-L1 and PD-L2), chemokines (CXCL9, CXCL10, and CXCL12), along with cytokines (TGF-β and IL-10), creates a complex immunosuppressive environment in EwS. This environment facilitates tumor growth and allows EwS to evade the host's immune response. Understanding these mechanisms is vital for developing targeted therapies that can enhance anti-tumor immunity in patients with EwS.

#### 3.2.1 Programmed death ligand-1 and -2 (PD-L1 and PD-L2)

PD-L1 and PD-L2 are ligands for PD-1 receptors in activated T cells. PD-L1 is expressed in significant proportion in EwS. A study reported that 39 % of EwS expressed PD-L1, highlighting its potential role in mediating immune evasion within this tumor type [51]. However, other studies show variable PD-L1 expression rates across different sarcoma, with EwS exhibiting lower levels of PD-L1 compared to other sarcomas like leiomyosarcoma [52]. PD-L2 expression has been less extensively studied in EwS, but it is known to play a similar role in immune modulation as PD-L1. Both ligands suggest a robust mechanism for tumors to evade immune detection [53], [54], [55], [56].

PD-1, when interacting with either PD-L1 or PD-L2, triggers the phosphorylation of tyrosine residues in the immunoreceptor tyrosine-based switch motifs (ITSM) and immunoreceptor tyrosine-based inhibitory motifs (ITIM) within the cytoplasmic region of PD-1 [57]. This phosphorylation recruits SHP-2, a phosphatase that dephosphorylates key signaling molecules associated with T-cell activation, such as CD3 and ZAP70. This leads to a reduction in the proliferation, production of cytokines, and cytotoxic function of T-cells, as shown in Fig. 3 **and** Table 1**.** This condition is known as T-cell exhaustion, which leads to a decrease in the capacity of T-cells to identify and eliminate tumor cells, thus leading to uncontrolled tumor growth [57], [58].

Taken together, unlike other cancers, EwS tumors show heterogeneous or low PD-L1 expression, limiting the effectiveness of PD-1 inhibitors. Combining anti-CXCR4 or anti-TGF-β with standard anti-PD-1 therapy may enhance anti-tumor immune response in EwS. While PD-L1 and PD-L2 contribute to immune evasion in EwS, their relative roles and interactions with other immunosuppressive pathways remain incompletely understood. Further studies exploring the interplay between EwS tumor cells, the tumor microenvironment, and PD-1 signaling may reveal new insights into immune resistance mechanisms and inform the development of more effective immunotherapies for this aggressive malignancy.

#### 3.2.2 Human leukocyte antigen G (HLA-G)

HLA molecules are expressed in EwS under inflammatory conditions, and their expression is crucial for tumor recognition by reactive T cells. Their loss can reduce tumor susceptibility to immunotherapies [17]. HLA has different receptors, such as immunoglobulin-like transcript 2 and 4 (ILT2/4) and killer immunoglobulin-like receptors (KIR) [17], [18]. It interacts with inhibitory receptors on T-cells, including KIR2DL4 and CD8, resulting in decreased T-cell proliferation and cytotoxic activity [18], as shown in Fig. 3. This interaction can induce apoptosis in activated CD8+T-cells, diminishing the anti-tumor immune response **(**Table 1**)** [24]. Human leukocyte antigen G functions as an “immune shield” by attaching to specific inhibitory receptors on immune cells like T-cells and NK cells [50]. Additionally, HLA-G binding triggers a range of immune suppression that helps cancer cells evade host immune monitoring [4]. HLA-G inhibits the maturation of dendritic cells, which is essential for effective T-cell priming and hinders the immune response against tumors [50]. It also facilitates the differentiation of naive T-cells into regulatory T-cells, which further suppresses anti-tumor immunity and supports tumor growth and metastasis **(**Table 1**)**.

HLA-G can drive macrophage polarization towards the M2 phenotype, marked by immunosuppressive functions that inhibit T-cell responses and advance tumor progression by releasing various growth factors [42], [47] **(**Fig. 3**)**. This molecule can also suppress NK cell activity, essential for identifying and eliminating tumor cells. Studies indicate that soluble forms of HLA-G can obstruct NK cell-mediated cytotoxicity against EwS cells [24]. HLA-G facilitates immune escape in EwS through several mechanisms that inhibit T-cell activation, alter dendritic cell function, encourage regulatory T-cell differentiation, and impair NK cell responses. Exploring these pathways presents potential therapeutic strategies to enhance treatment outcomes for EwS patients by targeting the immunosuppressive actions of HLA-G **(**Table 1**)** [4].

#### 3.2.3 Chemokines (CXCL9, CXCL10, CXCL12)

EwS cells secrete various chemokines to the tumor site that attract immune cells, particularly leukocytes. This recruitment can enhance tumor growth by creating a favourable microenvironment, although some immune cells may contribute to anti-tumor immunity [4]. In addition to anti-tumor immunity, chemokines play a dual role in angiogenesis (forming new blood vessels). For example, CXCL1 and CXCL2 promote angiogenesis [1], whereas CXCL9 and CXCL10 exhibit angiostatic effects **(**Table 1**)** [4], [59], which can influence the tumor's vascularization and growth. CXCL9 and CXCL10 were associated with increased tumor infiltrating CD8+T cells [14]. Chemokine receptors, particularly CXCR4, are associated with the metastatic behavior of EwS [60]. High levels of CXCR4 expression correlate with poor prognosis and increased metastasis, as it directs tumor cells toward sites rich in its ligand, CXCL12. This receptor-ligand interaction facilitates the migration of EwS cells to distant organs, particularly the lungs and bones, as illustrated in Fig. 3. Studies indicate that CXCR4 expression is notably higher in cell lines derived from metastatic tumors than those from localized tumors, suggesting its role as a biomarker for metastatic potential in EwS [60]. Furthermore, the transcription factor EWS-FLI1, characteristic of EwS, regulates the expression of both CXCR4 and CXCR7 (receptor involved in metastasis), highlighting its importance in tumor biology [22], [61], [62]. EwS cells utilize chemokines to manipulate their microenvironment strategically. Recruiting various immune cells to the tumor site can enhance growth through immunosuppression while influencing angiogenesis, as mentioned in Table 1. The interplay between different chemokines and their receptors affects local tumor dynamics and plays a crucial role in metastasis. Understanding these mechanisms provides insights into potential therapeutic strategies targeting chemokine signaling pathways to improve patient outcomes in EwS [7], [57].

#### 3.2.4 Immunosuppressive cytokines (TGF-β, and IL-10)

TGF-β is a versatile cytokine that plays a significant role in the pathophysiology of multiple cancers, including EwS. TGF-β signaling primarily functions through the canonical Smad and non-canonical pathways, encompassing MAPK and PI3K/AKT signaling [26], [45], [63]. In EwS, the disruption of these pathways has been linked to the development of tumors, as shown in Fig. 3 **and** Table 1. Specifically, TGF-β can initiate epithelial-to-mesenchymal transition (EMT), increasing cell motility and invasiveness, critical for cancer spread [26], [64]. When TGF-β binds to its receptors in the canonical pathway, SMAD proteins are activated and move to the nucleus to influence gene expression. EwS cells can inhibit the canonical pathway by EWS-FLI1, which lowers TGFBR2 expression and reduces tumor-suppressive functions [65], as shown in Fig. 3.

The non-canonical pathways include MAPK signaling (e.g., JNK and ERK), which can trigger either apoptosis or autophagy in response to TGF-β signaling [26]. During the initial stages of tumor development, TGF-β functions as a suppressor by inducing cell cycle arrest and apoptosis. It does this by increasing the expression of cyclin-dependent kinase (CDK) inhibitors, such as p21 and p15Ink4b, which halt cell proliferation [45], [66]. On the other hand, at advanced stages of cancer, elevated levels of TGF-β are produced by both tumor cells and the TME, facilitating immune evasion and boosting metastatic capability [45], [66]. This occurs through the promotion of EMT, enabling cancer cells to migrate and invade adjacent tissues, modulation of immune responses by inhibiting CD8+T-cell function and encouraging macrophage differentiation into an M2 phenotype that supports tumor growth, as well as stimulating angiogenesis and extracellular matrix remodeling that aid in tumor progression **(**Table 1**)**. The intricate role of TGF-β in EwS indicates that targeting this pathway might offer a viable therapeutic strategy. Recent studies suggest that reactivating TGF-β signaling could restrain EwS cell proliferation, underscoring its potential as a treatment target [67]. Additionally, neutralizing TGF-β might amplify anti-tumor immune responses, positioning it as a candidate for combination therapies to enhance patient outcomes.

IL-10 directly suppresses the activation and function of CD8+T-cells and NK cells. It diminishes their capacity to produce key effector cytokines such as IFN-γ and tumor necrosis factor-alpha (TNF-α), crucial for robust anti-tumor immunity [68]. Like TGF-β, IL-10 promotes the recruitment and activation of regulatory T-cells [69]. Elevated levels of IL-10 are often associated with increased numbers of Tregs within the TME, which further suppresses the immune response toward tumors **(**Table 1**)** [69]. IL-10 also plays a role in supporting tumor growth by creating an immunosuppressive microenvironment. It inhibits pro-inflammatory responses, and IL-10 helps maintain an environment that favors tumor survival, leading to poor prognosis as it allows EwS tumors to grow unchecked [4], [54], [57]. TGF-β and IL-10 are key immunosuppressive cytokines that play critical roles in EwS by inhibiting effector immune cell functions while fostering an environment that supports tumor growth and metastasis, as mentioned in Table 1. Targeting these cytokines or their signaling pathways presents a promising approach to enhance anti-tumor immunity and improve therapeutic outcomes for patients with this aggressive malignancy. Ongoing research into the mechanisms of how these cytokines operate will be essential for developing effective treatments aimed at counteracting their immunosuppressive effects in EwS [4], [54], [57].

## 4 Current advances and challenges in Ewing sarcoma therapy

EwS is an aggressive cancer requiring multimodal treatment, but survival rates for metastatic cases remain low. Relapses are mostly systemic, with poor five-year survival post-relapse (15–25 %). Standard chemotherapy regimens have limited efficacy, and while high-dose ifosfamide improves survival, it causes significant toxicity. These challenges highlight the need for more effective, less toxic treatments targeting EwS biology. The aberrant angiogenesis, a key process in tumor progression, has emerged as a promising target [70]. Tyrosine kinase inhibitors (TKIs) such as regorafenib and cabozantinib have demonstrated encouraging antitumor activity in preclinical models and early-phase clinical trials [70], [71], [72], [73]. While their efficacy as monotherapy is comparable to standard chemotherapy, ongoing studies are exploring their utility in combination therapies and maintenance strategies to enhance outcomes [71], [72]. Immunotherapy represents another promising avenue. Chimeric antigen receptor (CAR) T-cell therapies targeting specific markers such as GD2 and EGFR are being actively investigated, with clinical trials currently underway for EwS (NCT03635632, NCT03618381, NCT03373097, NCT03356782, [74]). Preliminary results from trials of GD2 CAR T-cells in neuroblastoma patients have shown significant activity, and similar success in EwS is eagerly anticipated [75]. Other strategies to enhance immune responses in Ewing sarcoma (EwS) include using CARs to boost NK cell activity, as demonstrated in phase I/II studies involving allogeneic haploidentical NK cell infusions combined with chemotherapy or radiotherapy. In addition, a phase I trial of memory T-cells expressing NKG2D recruits patients with refractory sarcoma (NCT05952310, NCT03495921). However, several other immunotherapeutic strategies are being explored to overcome tumor resistance and enhance immune system activation. For instance, immune checkpoint inhibitors aim to restore immune recognition of tumor cells by targeting pathways such as PD-1/PD-L1 and CTLA-4, with combination strategies being investigated to enhance their efficacy given the relatively low mutational burden of Ewing sarcoma and lack of PD-L1 expression in EwS tumors [4]. Multiple vaccines have advanced to phase III clinical trials for the treatment of Ewing sarcoma (NCT03495921, NCT00001566) [4]. Monoclonal antibodies, particularly those targeting IGF-1R, have reached phase III (NCT02306161) [4]. IGF-1R plays a crucial role in blocking tumor growth and survival and is being tested both as monotherapies and in combination with chemotherapy or other immunotherapies in EwS [76]. Oncolytic viruses represent another emerging approach, utilizing genetically engineered viruses to selectively infect and destroy cancer cells while triggering an anti-tumor immune response, with promising results observed in preclinical and early-phase clinical trials [9]. Additionally, cytokine-based therapies, including immune-stimulating molecules such as IL-2, GM-CSF, and interferon-gamma, are being explored to enhance immune activation and counteract the immunosuppressive tumor microenvironment in EwS [10].

Another promising approach under investigation is oral metronomic therapy, which modulates the immune response and inhibits angiogenesis. This strategy uses continuous low-dose chemotherapy over an extended period to disrupt tumor vasculature and limit the blood supply necessary for tumor survival and progression [77], [78], [79]. Moreover, metronomic therapy has been shown to enhance anti-tumor immune responses, potentially improving clinical outcomes in EwS patients [80]. Looking ahead, preclinical development of T-cell receptor (TCR)-transduced T-cells and bispecific T-cell engagers could further revolutionize the therapeutic landscape for EwS.

## 5 Conclusion, limitations, and future aspects

EwS is a type of bone or soft tissue cancer that primarily occurs in children and young adults. A combination treatment strategy is used even when the disease only appears to be localized at diagnosis [74]. Recent research has focused on innovative therapies targeting the EWSR1-FLI1 fusion protein, a hallmark of EwS, through approaches like PARP inhibitors and TKIs such as regorafenib and cabozantinib. A comprehensive approach integrating CAR-T cell therapy with other immunotherapeutic strategies, such as immune checkpoint inhibitors, cancer vaccines, monoclonal antibodies, oncolytic viruses, and cytokine-based therapies, may offer the most effective pathway for overcoming tumor resistance and improving outcomes in EwS. Our review methodology adheres to the PRISMA guidelines, ensuring a systematic search regarding immune infiltration in EwS. Ultimately, 20 peer-reviewed original research articles met the stringent inclusion criteria and were included in the review. These studies provide valuable insights into the interactions between immune infiltration, ECM, and TIME in EwS, enhancing understanding its immunobiology. The intricate interplay between tumor-infiltrating immune cells and the TME significantly influences the pathogenesis and therapeutic responses in EwS. The low occurrence of EwS makes it challenging to collect sufficient clinical samples for robust statistical analyses. Many studies are limited to small cohorts, which do not accurately represent the broader patient population. Although some studies have identified immune cell infiltration patterns, they often lack a detailed characterization of specific immune cell subsets within the tumor microenvironment. Such detail is crucial for understanding how different immune cells interact and contribute to tumor biology. Most research has focused on correlating immune cell frequencies with clinical outcomes without delving into the underlying mechanisms driving these correlations. New experimental models, such as humanized mice, are being developed. However, there is still a need for models that can effectively replicate the multifocal characteristics and spread patterns of EwS. There is limited knowledge regarding how various treatments (e.g., chemotherapy and radiation) affect the immune microenvironment in EwS.

Traditional immunotherapy targeting the PD-1/PD-L1 axis has shown limited success in treating EwS, primarily due to the tumor's unique immunological characteristics. Studies indicate that EwS cells generally do not express PD-L1 in their pre-therapeutic microenvironment. While they can upregulate this ligand under inflammatory conditions, this response is insufficient for effective immune activation against the tumor. The low overall immune infiltration in EwS, coupled with immunosuppressive factors within the TME, complicates the efficacy of PD-1 blockade strategies [8], [81]. In contrast, emerging research suggests that targeting the TME itself may offer a more promising therapeutic avenue, which is the motivation behind our review. By concentrating on the TME, we aim to improve the effectiveness of current immunotherapies and create novel strategies that may enhance outcomes for EwS patients.

## CRediT authorship contribution statement

**Rajiv Ranjan Kumar:** Writing – review & editing, Writing – original draft, Methodology. **Nikita Agarwal:** Writing – original draft. **Akshi Shree:** Writing – original draft, Supervision. **Jaya Kanta Gorain:** Writing – original draft. **Ekta Rahul:** Writing – review & editing, Visualization. **Shuvadeep Ganguly:** Writing – review & editing. **Sameer Bakhshi:** Writing – review & editing, Supervision, Resources, Funding acquisition. **Uttam Sharma:** Writing – review & editing, Visualization, Supervision, Resources, Funding acquisition, Conceptualization.

## Author contribution

**U.S.** and **S.B.** conceived the idea. **R.R.K.** and **U.S.** performed the literature screening. **R.R.K.** contributed to the writing of the methodology, the preparation of Fig. 1, and the section on current advances and challenges in Ewing Sarcoma therapy. **N.A.** was responsible for writing the sections on immune molecules in tumor microenvironment, conclusions, limitations, and future aspects, as well as preparing Fig. 3 and Table 1. **J.K.G.** contributed to writing the sections on tumor-infiltrating cells and the conclusion. **A.S.** wrote the introduction and the section on the implications of tumor-infiltrating cells and molecules in EwS and prepared Fig. 2. **E.R.** created the summary table of the included studies. **U.S.** provided overall supervision for the project. **S.G., U.S.** and **S.B.** revised the manuscript. All authors approved the final manuscript.

## Declaration of competing interest

The authors declare that they have no known competing financial interests or personal relationships that could have appeared to influence the work reported in this paper.
